# Compound Heterozygote of Point Mutation and Chromosomal Microdeletion Involving *OTUD6B* Coinciding with *ZMIZ1* Variant in Syndromic Intellectual Disability

**DOI:** 10.3390/genes12101583

**Published:** 2021-10-07

**Authors:** Tim Phetthong, Arthaporn Khongkrapan, Natini Jinawath, Go-Hun Seo, Duangrurdee Wattanasirichaigoon

**Affiliations:** 1 Division of Medical Genetics, Department of Pediatrics, Faculty of Medicine Ramathibodi Hospital, Mahidol University, Bangkok 10400, Thailand; t.phetthong@pcm.ac.th (T.P.); manualsibc@gmail.com (A.K.); 2 Division of Medical Genetics, Department of Pediatrics, Phramongkutklao Hospital and Phramongkutklao College of Medicine, Bangkok 10400, Thailand; 3 Program in Translational Medicine, Faculty of Medicine Ramathibodi Hospital, Mahidol University, Bangkok 10400, Thailand; jnatini@hotmail.com; 4 Integrative Computational Bioscience Center, Mahidol University, Nakhon Pathom 73170, Thailand; 5 Department of Medical Genetics, 3billion, Inc., Seoul 05505, Korea; ghseo@3billion.io

**Keywords:** intellectual disability, OTUD6B, ZMIZ1, chromosomal microdeletion

## Abstract

The OTUD6B and ZMIZ1 genes were recently identified as causes of syndromic intellectual disability (ID) with shared phenotypes of facial dysmorphism, distal limb anomalies, and seizure disorders. OTUD6B- and ZMIZ1-related ID are inherited in autosomal recessive and autosomal dominant patterns, respectively. We report a 5-year-old girl with developmental delay, facial phenotypes resembling Williams syndrome, and cardiac defects. The patient also had terminal broadening of the fingers and polydactyly. Cytogenomic microarray (CMA), whole exome sequencing (WES), and mRNA analysis were performed. The CMA showed a paternally inherited 0.118 Mb deletion of 8q21.3, chr8:92084087–92202189, with OTUD6B involved. The WES identified a hemizygous OTUD6B variant, c.873delA (p.Lys291AsnfsTer3). The mother was heterozygous for this allele. The WES also demonstrated a heterozygous ZMIZ1 variant, c.1491 + 2T > C, in the patient and her father. This ZMIZ1 variant yielded exon 14 skipping, as evidenced by mRNA study. We suggest that Williams syndrome-like phenotypes, namely, periorbital edema, hanging cheek, and long and smooth philtrum represent expanded phenotypes of OTUD6B-related ID. Our data expand the genotypic spectrum of OTUD6B- and ZMIZ1-related disorders. This is the first reported case of a compound heterozygote featuring point mutation, chromosomal microdeletion of OTUD6B, and the unique event of OTUD6B, coupled with ZMIZ1 variants.

## 1. Introduction

Multiple congenital anomalies and intellectual disabilities (ID) are common features of many chromosomal and single gene disorders. Owing to the phenotypic and genetic heterogeneity associated with congenital anomalies and ID, establishing a causative diagnosis, based on clinical signs and symptoms, can be challenging. Chromosomal microarray (CMA) has been recommended as the first-tier test in evaluating patients with unexplained ID and multiple congenital anomalies, as endorsed by the American College of Medical Genetics and Genomics (ACMG) [1,2]. CMA does not capture single nucleotide variations and small insertions/deletions (indels), so patients with negative CMA findings undergo next-generation sequencing (NGS), using either whole exome or whole genome sequencing (WES/WGS).

The *OTUD6B* (ovarian tumor domain-containing 6B) gene, located on chromosome 8q21.3 and consisting of 7 exons, encodes a 323-amino acid protein. OTU6B may play roles in the initiation of protein synthesis, repression of DNA synthesis, and regulation of cell growth and proliferation (uniprot.org/uniprot (accessed on 15 August 2021) [3,4]. In 2017, Autosomal recessive mutations of *OTUD6B* were first described to cause intellectual disability associated with seizure, dysmorphic features, and congenital malformations, including distal limb abnormalities. Some studies propose clinical similarities between *OTUD6B*-related phenotypes and Rubinstein-Taybi syndrome and Kabuki syndrome [5,6,7]. However, at present, phenotypic and genotypic data related to OTUD6B are still limited [5,6,7,8,9,10].

*ZMIZ1* (zinc finger MIZ-domain containing 1), mapped to 10q22.1-q22.3 and containing 21 exons, encodes a 1067-amino acid peptide. ZMIZ1 protein functions as a transcriptional regulator that interacts with other proteins involved in the development of neuronal and glial cells [11]. Heterozygous *ZMIZ1* variants have been found to be associated with neurodevelopmental disorder with dysmorphic facies and distal skeletal anomalies (NEDDFSA), including infantile hypotonia, seizures, and ID of varying severity. Since the first description of *ZMIZ1* as the cause of ID, in 2019, only a handful of cases have been reported, most of which represented de novo occurrences; only two families were reported with inherited mutations [11,12].

Herein, we describe novel genetic defects of *OTUD6B*, 8q21.3 microdeletion compounded with a point mutation of the gene, in a patient with ID and Williams syndrome-like phenotypes. We also identify a novel inherited *ZMIZ1* variant, resulting in a splicing error. Similarities and differences of clinical manifestations, caused by mutations of the two genes, are also discussed.

## 2. Materials and Methods

### 2.1. Patients

The patient was a 5-year-old girl who was referred for further genetic investigation owing to her developmental delay, cardiac, and hand anomalies, as well as suspected Williams syndrome (OMIM 194050). Prior investigation included karyotype and fluorescence in situ hybridization (FISH) analysis of 7q11.23, which demonstrated 46, XX, while no deletion was detected by the FISH study.

### 2.2. Cytogenomic Microarray

DNA was extracted from peripheral blood, using Gentra Puregene kit (QIAGEN Hilden, Germany). A single nucleotide polymorphism array (Illumina Infinium CytoSNP-850 K Beadchip, Illumina, San Diego, CA, USA) was performed and analyzed using BlueFuse Multi software v4.1 (Illumina). The Database of Genomic Variants (DGV) and the Thai CNV database [13] were used to exclude common structural variations in the Thai population.

In an attempt to enroll additional patients into the study, we reviewed curated databases, ClinGen and DECIPHER, searching for patients described as having small (~5Mb or less) microdeletions of the 8q21.3 region.

### 2.3. Whole Exome Sequencing Analysis

Whole exome sequencing (WES) was performed. All exons of all human genes (~22,000) were captured by Twist Human Core Exome Kit (Twist Bioscience, San Francisco, CA, USA). The captured genomic regions were sequenced using NovaSeq 6000 (Illumina). The raw genome sequencing data analysis, including alignment to the reference sequence, was found (original GRCh37 from NCBI, February 2009, Figure S1). Mean depth of coverage was 100× (>10× = 99.2%). The variant calling, annotation, and prioritization were performed as previously described [14]. The variants with minor allele frequency (MAF) > 0.05 were filtered out based on data in a population database (gnomad.broadinstitute.org (accessed on 14 April 2020)) and in accordance with the 2015 guidelines of the American College of Medical Genetics and Genomics and the Association of Molecular Pathology (ACMG/AMP) [15]. The human phenotype ontology (HPO) terms: global developmental delay (HP:0001263); seizure (HP:0001250); postaxial hand polydactyly (HP:0001162) were accessed to measure the similarity with each of ~7000 rare genetic diseases [16,17]. The pathogenicity of the identified variants was determined in accordance with the literature and disease databases, including ClinVar (ncbi.nlm.nih.gov/clinvar (accessed on 14 April 2020)), UniProt (uniprot.org (accessed on 15 August 2021)), and the ACMG/AMP guidelines [15].

The presence of the variants identified in the studied individuals was confirmed by Sanger sequencing. Details of the primers used are provided in the Supplementary data (Table S1). Segregation of the variant(s) and the clinical phenotypes was also evaluated.

### 2.4. mRNA Study of ZMIZ1 Mutation

Total mRNA was extracted from peripheral blood specimens of the studied individuals using Invitrogen TRIzol reagent (ThermoFisher Scientific, Waltham, MA, USA), followed by reverse transcription, using iScript Reverse Transcription Supermix for RT-qPCR (Bio-Rad, Hercules, CA, USA), in accordance with the manufacturer’s instructions. PCR and sequencing of the cDNA were performed using primers specific for exons 12 and 17 of the ZMIZ1 gene as follows: forward, 5′-ACCCTGGAGAGCCCAACTAT-3′ and reverse, 5′-GGTCAGACCTCCACATCAGC-3′. The PCR products representing normal and aberrant transcripts were cut out from the agarose gel, then purified using QIAquick Gel Extraction Kit (QIAGEN), before being subjected to Sanger sequencing.

Primers were designed using PRIMER3 (frodo.wi.mit.edu (accessed on 9 September 2020)). The reference sequences were NC_000008.10 and NM_016023 for OTUD6B study, as well as NC_000010.10 and NM_020338.3 for ZMIZ1 analysis.

This study was approved by the Ramathibodi Hospital Human Research Ethics Committee (COA MURA2020/1273). Genetic analyses were performed following the provision of written informed consent. Consent for the publication of identifiable data of the patient, including photograph, was provided by the parents.

## 3. Results

### 3.1. Clinical Manifestations and Family Data

The patient was born by cesarean section at a gestational age of 37 weeks, with Apgar scores of 9 and 10 at 1 and 5 min, respectively. Her birth parameters were normal, including weight of 2800 g (50th centile), length of 50 cm (50th–75th centile), and head circumference of 31 cm (3rd–10th centile). She was noted to have respiratory distress right after birth, and was confirmed to have atrial septal defect, ventricular septal defect, and mild pulmonic stenosis. The patient underwent surgical closure of atrial septal defect at 12 months.

As an infant, the patient exhibited failure to thrive. At 18 months of age, her growth parameters were at the lowest normal limit, including weight of 8.4 kg (3rd–10th centile), height of 75.5 cm (3rd centile), and head circumference of 44 cm (3rd centile). Her physical growth gradually improved with age, and it has been within the normal range since the age of 3 years.

Developmentally, the patient exhibited developmental delay. She could hold to stand up at 18 months, started walking at 24 months, walked independently at 3.6 years, walked up and down stairs at 4 years, and jumped and hopped by 6 years. At 4 years, she was noted to walk on her tiptoe, but without lower limb spasticity. As for language development, she spoke a few single words at 30 months, put 2–3 words together at 3 years and talked in short phrases by 5 years. The patient was able to start toilet training and took part in showering, dressing, and feeding herself at 4 years of age.

The patient first developed generalized tonic-clonic (GTC) seizures at 12 and 24 months, preceded by high-graded fever. The third (at 28 months) and fourth episodes (at 29 months) were atonic and tonic-clonic seizures, respectively, with no precipitating cause identified. Her seizures were controlled by phenobarbital, but the medication was discontinued soon after, owing to cutaneous drug reaction, leading to multiple episodes of seizures. Sodium valproate was introduced and controlled her seizures well. At the age of 7 years, there were two episodes of breakthrough seizures, leading to the initiation of topiramate and tailing of valproate.

**Figure 1:**
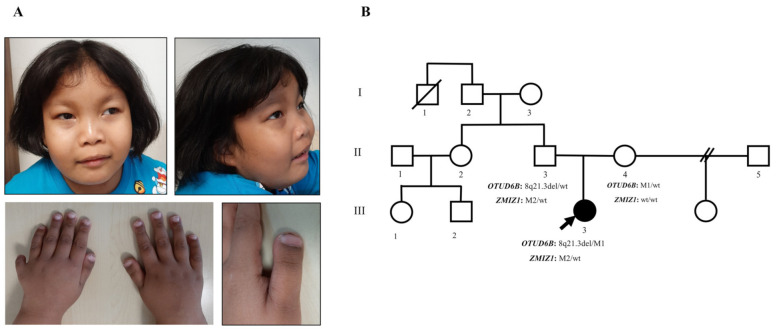
Clinical features and pedigree. (**A**) Photograph of the patient at 5 years of age, showing periorbital edema, arched and sparse lateral eyebrows, prominent nasal bridge, long and smooth philtrum, thin vermillion of the upper lip, hanging lower cheek, postaxial polydactyly of the left hand, terminal broadening of fingers, and broad thumbs. (**B**) Pedigree of the present family. The affected individual is shown by the black-filled symbol. An arrow indicates the proband. The OTUD6B and ZMIZ1 genotype of each individual is indicated below the corresponding symbols. M1: c.873delA; M2: c.1491 + 2T > C; wt: wild type.

Physical examination at 5 years of age revealed arched and sparse lateral eyebrows, periorbital puffiness, prominent nasal bridge, long philtrum, thin vermillion of the upper lip, hanging cheek, postaxial polydactyly at the left hand, terminal broadening of the fingers, and broad thumbs and great toes (Figure 1A). Neurological examination demonstrated normal muscle tone and deep tendon reflex, no spasticity, but a tight heel cord.

Other investigations revealed normal hearing. Ophthalmological examinations at 5 years revealed normal visual acuity and fundoscopic examination, as performed by an ophthalmologist. Ultrasonography of the kidney and urinary system showed normal findings.

Family history was negative for consanguinity, seizures, developmental/intellectual problems, and congenital anomalies (Figure 1B). The patient’s father was healthy and did not have dysmorphic features or cardiac murmur, as examined by clinical geneticists (TP and DW). The father and his sister completed a university degree without difficulty.

At the time of this writing, the patient is 7.5 years old, weighs 41.4 kg (>97th centile), is 120 cm tall (50th centile), and has a head circumference of 52 cm (75th–90th centile). She communicates in short sentences with good comprehension, being better at the receptive than at the expressive part. She is active in daily life and attends kindergarten.

### 3.2. Identification of 8q21.3 Microdeletion Compounded with a Point Mutation Involving OTUD6B

**Figure 2:**
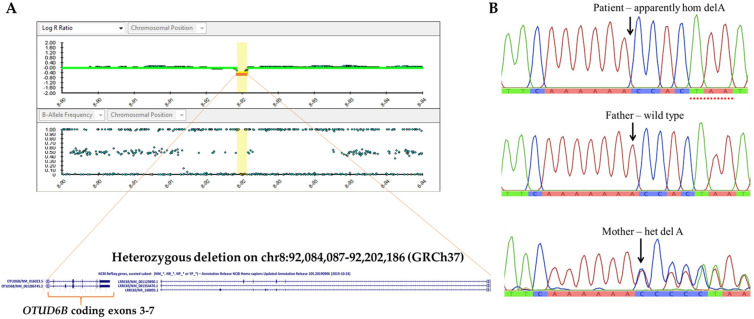
*OTUD6B* chromosomal and nucleotide mutations. (**A**) CytoSNP-850 K array showing Log R ratio (LRR) plot and B-allele frequency (BAF) plot and revealing a 0.118 Mb deletion of chromosome 8q21.3. (**B**) Genomic DNA sequence of exon 6 showing delA in the patient and her father.

CMA analysis of the patient revealed a 118,100 bps deletion of chromosome 8q21.3 (Chr8: 92,084,087–92,202,186, GRCh37) (Figure 2A). The deleted fragment encompassed 2 genes: OTUD6B and LRRC69, and led to a deletion of exons 3–7 of OTUD6B. Subsequent parental CMA studies indicated that the father also harbored the 0.118 Mb deletion. This deletion was classified as a variant of uncertain significance (VUS) based on the following criteria: a CNV which contains a small number of genes, but it is not known whether the genes in the interval are dosage sensitive (category 3A); a CNV within an individual gene (category 2E, deletion metric, and 2I, duplication metric) with an unclear effect on the transcript reading frame [18]. Because OTUD6B is associated with autosomal recessive ID, with characteristic phenotypes that matched our patient’s manifestations, we believed that there could be another OTUD6B mutation in compound with the 8q21.3 microdeletion that is responsible for the patient’s phenotypes. Therefore, we performed singleton WES.

The WES analysis of the patient’s specimen identified a hemizygous variant, c.873delA, in exon 6 of the OTUD6B gene (NM_016023). The deletion variant was predicted to cause a frameshift mutation and premature protein truncation (p.Lys291AsnfsTer3). This allele (rs 762934606) is present at an extremely low frequency (0.00003926) in gnomAD (gnomad.broadinstitute.org (accessed on 14 April 2020)) and was classified as likely pathogenic [15]. The mother was found to be heterozygous for this allele, as confirmed by Sanger sequencing (Figure 2B).

The segregation of biallelic variants (in trans) of OTUD6B in the patient, and the heterozygous state in the asmptomatic parents, suggests that the 8q21.3: 0.118 Mb deletion and the OTUD6B:c.873delA each respresent a pathogenic recessive allele. A review of curated databases disclosed small 8q21.3 microdeletion, involving OTUD6B in one case, listed in ClinGen, and three cases in DECIPHER. The sizes of the deletions ranged from 3.597 to 5.781 Mb. The phenotypes and genomic locations of these patients were detailed in the Supplementary data (Table S2). These patients could potentially be candidates for the study of genetic compounds between chromosomal structural defect(s) and OTUD6B point mutation. However, we failed to reach these cases.

### 3.3. ZMIZ1 Splicing Mutation and its Effect on the mRNA Transcription

**Figure 3:**
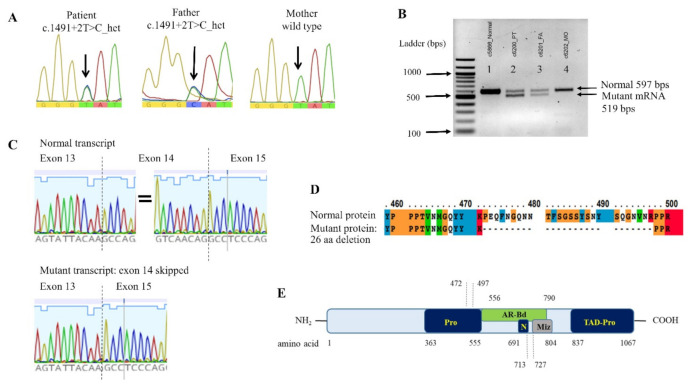
*ZMIZ1* genomic and mRNA analysis. (**A**) Genomic DNA sequence of exon 14 identifying heterozygous T > C variant in the proband and the father. (**B**) mRNA (cDNA) product of *ZMIZ1* exons 12–17, shown on 2% agarose gel. The normal control (lane 1) and the mother (MO, lane 4) exhibit only normal sized 597-bp product, whereas the patient (PT, lane 2) and the father (FA, lane 3) show both normal (597 bp) and aberrant (519 bp) mRNA products. (**C**) Sequence of the normal mRNA transcript and the mutant transcript with exon 14 skipped. (**D**) Clustal sequence alignment of the wild type and the mutant ZMIZ1 proteins. Noted is an in-frame deletion of 26 amino acids. (**E**) Partial schematic diagram of ZMIZ1. Noted is the location of the 26-aa deletion (aa 472–497) in proline-rich region (Pro). AR-Bd, primary binding region for androgen receptor; N, nuclear localization signal; Miz, Miz domain; TAD-Pro, transcriptional activation domain (proline-rich).

Unexpectedly, the WES analysis disclosed a heterozygous nucleotide substitution at the splice donor site of intron 14 of the ZMIZ1 gene, c.1491 + 2T > C. This variant was not present in gnomAD (MAF = 0) or T-REx: Thai Reference Exome Database (trex.nbt.or.th (accessed on 18 September 2021)) (MAF = 0) and was categorized as pathogenic (PVS1, PM2, PP3), based on the ACMG/AMP guidelines [15]. Sanger sequencing confirmed the heterozygous state of this allele in the patient and her father, along with the presence of a wild-type allele in the mother (Figure 3A).

The splicing error of c.1491 + 2T > C mutation was evaluated using in silico analysis software, NNSPLICE 0.9 (fruitfly.org/seq_tools/splice.htm (accessed on 9 September 2020)), which indicated the loss of splicing ability, with the score changed from 0.99 to 0. PCR of the mRNA transcript of coding exons 12–17 yielded both normal sized and aberrant transcripts in the specimens of the patient and her father, but it only yielded normal transcripts in her mother and a control individual (Figure 3B). Sanger sequencing confirmed the lack of the entire sequence of exon 14 in the mutant transcript (Figure 3C).

### 3.4. Clinical Similarities and Differences, Comparing between OTUD6B- and ZMIZ1-Related Phenotypes

Given clinical similarities of the phenotypes, caused by biallelic mutations of *OTUD6B* and heterozygous *ZMIZ1* mutations, and that the present patient harbored genetic defects of these two genes, we compared the phenotypes of the present patient to those in previous reports, as summarized in Table 1 [5,6,7,8,9,10,11,12].

We noticed that both *OTUD6B*- and *ZMIZ1*-related disorders share nonspecific phenotypes of ID, global developmental delay, hypotonia, microcephaly, feeding difficulty, and distal limb anomalies in at least two-thirds of the patients. However, some features were relatively prevalent and more specific to those of *OTUD6B*-linked ID, including substantial epilepsy, characteristic facial dysmorphisms, arched eyebrows/long palpebral fissures, prominent nasal bridge; broad thumb with/without polydactyly (postaxial). In contrast, facial features and distal limb anomalies found in *ZMIZ1*-related ID were nonspecific, while ophthalmological abnormalities were more frequently observed in this group.

## 4. Discussion

In this study, we demonstrated, for the first time, a genetic compound between a point mutation and a chromosomal structural variant of 8q21.3 as the cause of OTUD6B-linked disorder. We also revealed the unique event of biallelic-OTUD6B mutations coincidental with a ZMIZ1 variant with potential pathogenicity. Both OTUD6B variants and the ZMIZ1 variant were inherited alleles and were not previously reported.

Although a genetic compound between a chromosomal defect and a nucleotide change is a rare cause of an autosomal recessive disorder [19], this possibility should be taken into consideration when working with patient(s) with complex conditions. Clinical geneticists should always determine the clinical correlation when dealing with VUS CNVs reported in such case. The present case emphasizes the importance of reviewing phenotypes associated with autosomal recessive gene(s) included in the rearranged chromosomal segment, regardless of the size of CNVs and their inheritance from an asymptomatic parent.

The hemizygous c.873delA, in exon 6 of the patient, was due to missing exons 3–7, as part of the 8q21.3 deletion of the homologous chromosome.

Nine mutations of OTUD6B have been reported in 12 families, all of them were from Hispanic origin or were from Mediterranean countries [5,6,7,8,9,10]. Our patient represents the first case from Southeast Asia, which could expand the facial phenotypes of this rare syndrome, from a diverse population. We used a facial recognition software (Face2Gene: FDNA Inc. Boston, MA, USA) to analyze the patient’s photograph; however, it yielded low gestalt meter, with top 5 suggested disorders including Turner syndrome, Prader-Willi syndrome, 22q11.2 deletion syndrome, α-thalassemia/mental-retardation syndrome, and Angleman syndrome. Therefore, we used the presumptive diagnosis of Williams syndrome, owing to the presence of periorbital edema and full cheek of this patient, while searching for an additional diagnosis through extensive genetic tests. The current study shows that dysmorphic features, including periorbital edema and hanging cheek resembling Williams syndrome, can be part of the facial gestalt of OTUD6B-related ID. This extends the list of overlapping syndromes of this rare disorder, in addition to Kabuki syndrome and Rubinstein–Taybi syndrome [7,10]. These findings support the notion that different ethnic backgrounds could affect the overall craniofacial appearance of individuals, both normal and those affected with teratogenic/genetic disorders [20,21].

Notably, a recurrent nonsense mutation of OTUD6B, c.433C > T (p.Arg145*), has been reported to be consistently associated with severe phenotypes, namely severe ID, restricted ambulation, and absent speech [5,7,9]. In contrast, a single family with homozygous missense mutation (p.Tyr216Cys) appeared to have mild to moderate ID, spared speech, and gross motor functions [5]. Patients including ours, with homozygous or compound heterozygous of null allele, due to small indels (c.469_473delTTAAC; c.873delA) [5], splicing variants (c.173-2A > G; c.324 + 1G > C/c.405 + 1G > A) [5,10], or partial gene deletion (exons 3–7 deletion) could display variable, mild-to-severe, phenotypes. The differences in clinical severity, due to the small indels and splicing mutations, may be influenced by various factors including the activation of nonsense-mediated mRNA decay, leaky splicing, modifier genes, and the presence of other, as yet unidentified factors, including epigenetics and coincidental disorder(s).

Homozygous Otud6b knockout mice were shown to have low viability, or die shortly after birth, and exhibited growth restriction and congenital heart and hand anomalies, similar to those observed in association with disruption of its human homologue [5].

OTUD6B is a deubiquinating enzyme which reverse the ubiquitination, a process of post-translational protein modification, thus maintaining the homeostasis of cellular functions. This gene is universally expressed in human tissue, including fetal brain and B-and T-cell lineages (uniprot.org/uniprot (accessed on 15 August 2021)). OTUD6B has been experimentally confirmed or predicted to interact with many genes, most of which are involved in cell cycle regulation and apoptosis. Given the global expression of OTUD6B, mutations of this gene could lead to abnormalities of multiple tissues and organs. However, the reasons for some specific findings, such as ID, seizures, intrauterine growth restriction, and hand and cardiac defects, as well as the mechanisms behind them, have remained unclear. Nonetheless, the involvement of proteasome assembly defect has been strongly suggested.

The PTK2 gene is one of the top 25 genes interacting with OTUD6B, as described in the protein-protein interaction database, STRING (string-db.org (accessed on 15 August 2021)). PTK2 is a non-receptor protein kinase that plays roles in early embryonic development, placental development, cardiomyocyte migration, normal heart formation, axonal growth, synapse formation, and neuronal cell migration. It is possible that some of the OTUD6B-related features are partly a result of defective interaction between the OTUD6B and PTK2 genes.

ZMIZ1 belongs to a group of transcriptional coregulators, the Protein Inhibitor of Activated STAT (PIAS)-like family. It regulates the functions of DNA-binding transcription factors, but it does not directly bind DNA [22]. ZMIZ1 directly interacts with NOTCH1, which plays critical roles in brain formation and neuronal and glial cell differentiation [23,24]. ZMIZ1 is also involved in the regulation of postmitotic positioning of pyramidal neurons in the developing cerebral cortex [11].

To date, 15 pathogenic, or likely pathogenic, ZMIZ1 variants have been described in 18 families, most of which represent de novo events. Two families were reported with dominantly inherited mutations and intra-familial variability [11,12].

Our patient inherited the ZMIZ1 splice variant, c.1491 + 2T > C, from her asymptomatic father (II-3). As specimens from the paternal relatives were not available for analysis, we could only guess that this allele could have been inherited through one of the paternal grandparents (I-2 and I-3) and might have been passed on to their other child (II-2) and grandchildren (III-1 and III-2).

We showed that the ZMIZ1 c.1491 + 2T > C allele resulted in a mutant transcript lacking the entire exon 14. It was predicted to yield a mutant protein with an in-frame deletion of 26 amino acids (aa 472–497; Figure 3D,E). The deleted fragment is limited within the proline-rich region and does not involve the transcriptional activation and the nuclear localization domains [11]. Therefore, it is possible that the altered protein does not exhibit significantly disrupted function, regarding transcriptional activation and its interactions with the NOTCH1.

We hypothesize that, in the present family, the c.1491 + 2T > C allele could be either non-pathogenic or pathogenic with incomplete penetrance and/or variable expression. Plausible mechanisms, underlying the reduced penetrance, include the status as an in-frame deletion outside the transactivation and nuclear localization domains, which are critical regions for its transcriptional activation functions; leaky splicing resulting in differential in vivo transcriptions contrasting to that seen in the in vitro experiments; other, as yet unidentified, factors such as modifier genes and genetic redundancy.

Notably, rare ZMIZ1 variants, in proximity to c.1491 + 2T > C allele, have been described as follows: 1 individual with c.1491 + 1G > A (South Asian); 5 individuals with c.1491 + 2T > A (Latino/Admixed American) in gnomAD. The c.1491 + 1G > A was assigned as pathogenic (PVS1, PM2, PP3), whereas c.1491 + 2T > A was judged as benign (PVS1, PP3, BS1, BS2) based on the ACMG/AMP classification. Functional studies of these variants in animal and cellular models could further elucidate their true pathogenic effects.

Taking these findings together, we assume that the OTUD6B mutations were primarily responsible for the phenotypes of our patient because they cause an autosomal recessive disorder that is generally not associated with reduced penetrance. However, it is difficult to entirely rule out the effect of the ZMIZ1 variant, which might be involved in the the patient’s manifestations. The patient’s phenotypes better match OTUD6B-linked characteristics, namely, epilepsy, prominent nasal bridge, arched eyebrows, and broad thumb/polydactyly. Additionally, the father and his blood relatives (potential heterozygous for the ZMIZ1 variant) were asymptomatic.

## 5. Conclusions

In conclusion, we suggest that Williams syndrome-like phenotypes, namely, periorbital edema, hanging cheek, and long and smooth philtrum represent an expanded range of phenotypes of OTUD6B-related ID. Our data widen the genotypic spectrum of the OTUD6B- and ZMIZ1-related disorders. Clinical correlation and review of the phenotypes associated with the autosomal recessive gene(s) involved in the rearranged chromosomal segment should not be overlooked when dealing with VUS CNVs in patient(s) with an unrecognizable disorder.

## Figures and Tables

**Table 1:** Comparison of clinical characteristics of the present patient and those of *OTUD6B*- and *ZMIZ1*-related intellectual disability.

Clinical Characteristics	This Study (*n* = 1)	*OTUD6B*							*ZMIZ1*		
		Santiago-Sim et al. [5]	Straniero et al. [10]	Sánchez-Soler et al. [9]	Alkuraya et al. [6]	Romero-Ibarguengoitia et al. [7]	Abdel-Salam et al. [8]	Frequency	Carapito et al. [11]	Latchmanet al. [12]	Frequency
		(*n* = 12)	(*n* = 1)	(*n* = 1)	(*n* = 1)	(*n* = 1)	(*n* = 5)	(*n* = 21)	(*n* = 19)	(*n* = 3)	(*n* = 22)
**Central nervous system**											
Intellectual disability	+	12	+	+	NA	+	5/5	20/20 (100%)	19	3/3	22/23 (100%)
Epilepsy	+	12	+	+	++	+	3/4	19/20 (95%)	3/18	−	3/18 (16.7%)
Hypotonia	−	9	+	+	NA	+	NA	12/15 (80%)	10/16	2/3	12/19 (63.2%)
Motor delay	+	9	+	NA	+	+	5/5	17/20 (85%)	12/16	2/2	14/18 (77.8%)
Speech delay	+	9	+	+	NA	+	3/5	15/20 (75%)	15/17	3/3	18/20 (90%)
CNS anomalies	−	6	−	+	NA	+	3/4	11/19 (57.9%)	7/14	−	7/17 (41.2%)
Microcephaly	−	4	+	+	+	+	4/4	12/20 (60%)	8/10	1/3	9/13 (69.2%)
**Facial dysmorphism**											
Ptosis	−	1	+	+	+	−	2/5	6/21 (28.6%)	3/17	3/3	6/20 (30%)
Downslant pf.	−	2	−	−	−	−	−	2/21 (9.5%)	2/18	1/3	3/21 (14.3%)
Long pf.	−	6	−	+	NA	+	NA	8/15 (53.3%)	1/19	−	1/22 (4.5%)
Arched eyebrows	−	3	−	+	+	+	NA	6/16 (37.5%)	1/19	−	1/22 (4.5%)
Prominent nasal bridge	+	5	−	−	+	+	NA	7/16 (43.8%)	2/19	−	2/22 (9.1%)
Long philtrum	+	6	+	+	+	+	4/5	14/21 (66.7%)	2/19	−	2/22 (9.1%)
Thin vermillion of upper lip	+	5	+	+	+	+	1/5	10/21 (47.6%)	−	1/3	1/22 (4.5%)
Micro/retrognathia	−	2	−	NA	+	−	−	3/20 (15%)	3/19	2/3	5/22 (22.7%)
Ear anomalies	−	7	+	+	+	+	3/5	14/21 (66.7%)	4/19	1/3	5/22 (22.7%)
**Congenital heart disease**	+	4	+	−	+	+	1/3	8/19 (42.1%)	4/17	2/3	6/20 (30%)
**Gastrointestinal system**											
Feeding difficulties	+	9	+	+	+	+	NA	13/16 (81.3%)	9/17	2/2	11/19 (57.9%)
Constipation	−	2	NA	+	NA	+	NA	4/14 (28.6%)	NA	NA	NA
**Skeletal system**											
Distal limb anomalies											
Broad thumbs	+	6	+	+	+	−	5/5	14/21 (66.7%)	−	−	−
Polydactyly	+	−	−	−	+	+	−	2/21 (9.5%)	−	−	−
Others ^a^	−	9	−	+	−	−	−	10/21 (47.6%)	12/18	-	12/21 (57.1%)
Sacral dimple	−	2	−	−	NA	+	1/5	4/20 (20%))	−	−	−
Scoliosis	−	5	−	−	−	+	NA	6/16 (37.5%)	−	−	−
**Endocrine system**											
Hypothyroidism	−	2	NA	NA	NA	+	−	3/18 (16.7%)	NA	NA	NA
**Opthalmologic abnormalities**	−	NA	+ ^b^	+ ^c^	−	−	(2/5) ^d^	2/9 (22.2%)	10/17 ^e^	1/3 ^f^	11/20 (55%)

+, present; −, absent; NA, not available; pf, palpebral fissure. ^a^ syndactyly, brachydactyly, overriding toes, arachnodactyly, clinodactyly; ^b^ Duane syndrome (restricted horizontal eye movement); ^c^ strabismus; ^d^ retinal degeneration due to a coincidental disorder caused by homozygous *RP1L1* mutation, therefore these two patients were not included in calculation of the frequency; ^e^ glaucoma (1), refractive errors/amblyopia (7), Duane syndrome (1), retinal coloboma (1); ^f^ myopia.

## Supplementary Materials

The following are available online at https://www.mdpi.com/article/10.3390/genes12101583/s1, Table S1: Primer sequences used in the present study, Table S2: List of patients with 8q21.3-8q22.1 microdeletion (size ≈ 5 Mb or smaller) involving OTUD6B, Figure S1: UCSC genome browser (GRCh37) showing structural variants of 8q21 in various databases.
