# Supplementary material for: Compound Heterozygote of Point Mutation and Chromosomal Microdeletion Involving OTUD6B Coinciding with ZMIZ1 Variant in Syndromic Intellectual Disability
Source: Genes (Basel). 2021 Oct 7;12(10):1583. doi: 10.3390/genes12101583 (PMC8535745; doi:10.3390/genes12101583)
Supplement: Supplementary file 1 [file genes-12-01583-s001.zip › genes-1380642-supplementary.pdf]

Table S1. Primer sequences used in the present study.

Name	Direction	Direction	Product size (bps)	Annealing temperature (°C)
Primers for genomic DNA study				
OTUD6B_E6_F	Forward	5′-GTTGCCCGTTCACACATATT-3′	533	60
OTUD6B_E6_R	Reverse	5′-AACCCTGCATGCTGTATTTC-3′		
ZMIZ1_F	Forward	5′-CGCCCTGCTCACTCAATATAC-3′	437	60
ZMIZ1_R	Reverse	5′-CTAGCCAGGCTGACACTTTAC-3′		
Primers for cDNA study				
cZMIZ1_F	Forward	5′-ACCCTGGAGAGCCCAACTAT-3′	597	60
cZMIZ1_R	Reverse	5′-GGTCAGACCTCCACATCAGC-3′		

OTUB6B reference sequences: NCBI, NC_000008.10, NM_016023, GRCh37
ZMIZ1 reference sequences: NCBI, NC_000010.10, NM_020338.3, GRCh37

Table S2. List of patients with 8q21.3-8q22.1 microdeletion (size ≈ 5 Mb or smaller) involving *OTUD6B*.

Database/source	Patient ID	Location (GRCh37) start-end	Deletion size (Mb)	Phenotype/Karyotype	Inheritance	Number of genes involved	*OTUD6B* deletion
This study	–	92084087–92202186	0.118	DD, CHD (ASD, VSD, PS), seizure, periorbital edema, prominent nasal bridge, long philtrum, thin vermillion of the upper lip, hanging cheek, postaxial polydactyly at the left hand, clubbing of fingers without cyanosis and broad thumb and great toes / 46, XX	paternally inherited	2	partial (exon 3–7)
ClinGen (ISCA)	nssv 578285	91953224–95550571	3.597	DD and additional significant morphological phenotypes [a] / NA	de novo	31	complete
DECIPHER	295608	87925926–93707150	5.781	ID, CHD (VSD, ASD), behavioral abnormality, blepharophimosis, depressed nasal bridge, epicanthus, external ear malformation, flat occiput, generalized hypotonia, Inguinal hernia, protruding tongue, sandal gap, upslanted palpebral fissure, wide mouth, wide nasal bridge / 46, XX	de novo	37	complete
DECIPHER	248172	91797901–97081252	5.283	Abnormality of the nasal septum, abnormality of the pinna, ankyloglossia, hypertelorism, hypospadias, joint laxity, pes planus, scoliosis, ulnar deviation of finger, umbilical hernia, upslanted palpebral fissure / 46, XY	de novo	43	complete
DECIPHER	2399	91953224–95550571	3.597	ID, CHD (PS), prominent nasal bridge, upslanted palpebral fissure, aged 23-years / 46, XX	unknown	31	complete

ASD, atrial septal defect; CHD, congenital heart disease; DD, developmental delay; ID, intellectual disability; NA, not available; PS, pulmonic stenosis; VSD, ventricular septal defect

[a] No detailed morphological phenotypes

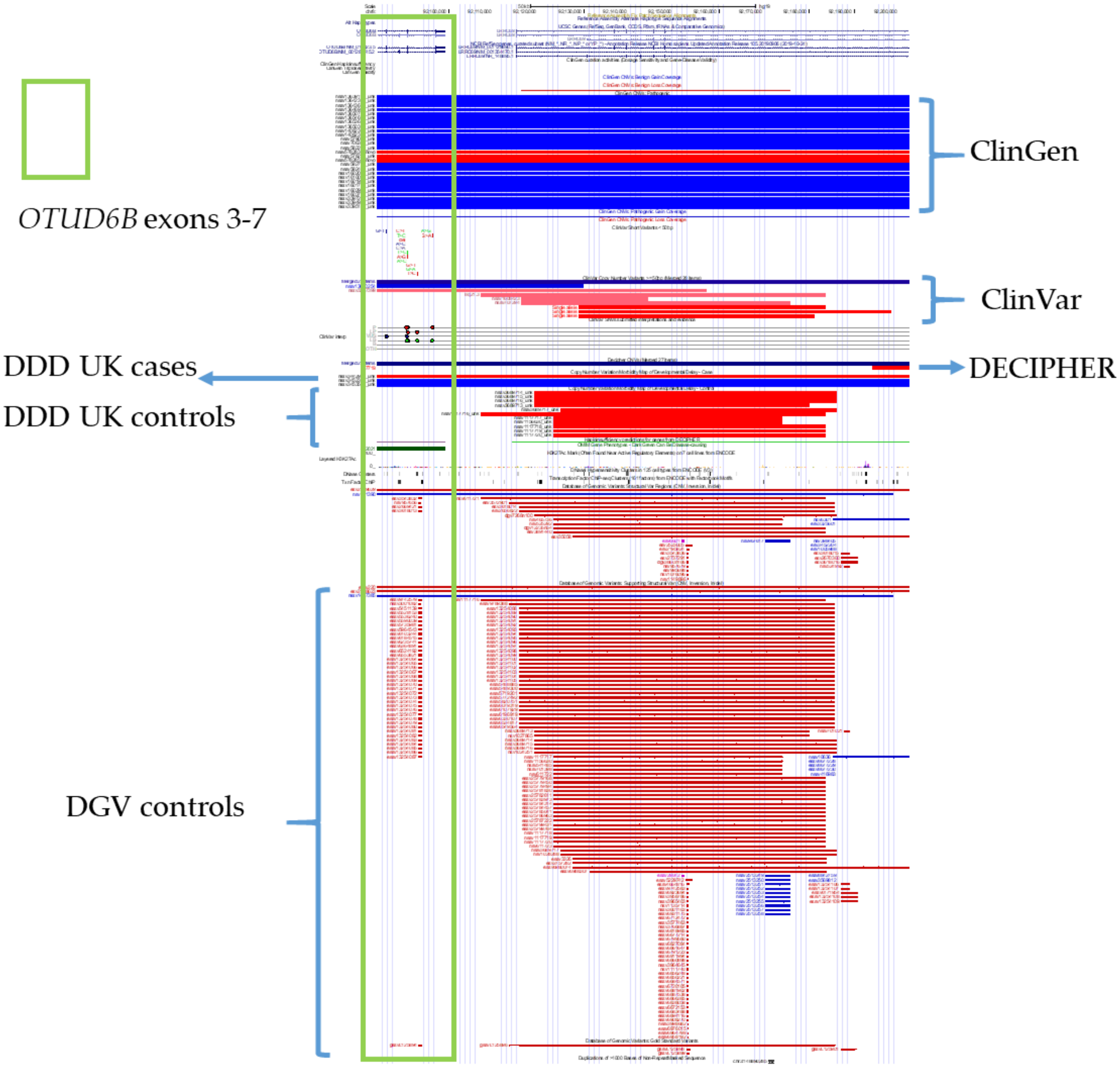

Figure S1. UCSC genome browser (GRCh37) showing structural variants of 8q21 in various databases. Blue indicates gain and red indicates loss. Noted the absence of *OTUD6B* deletion in DDD UK controls and DGV controls; three and one individuals with small 8q21 microdeletion involving *OTUD6B* in ClinGen and DECIPHER databases, respectively.
